# Photocatalytic, Morphological and Structural Properties of the TiO_2_-SiO_2_-Ag Porous Structures Based System

**DOI:** 10.3390/ma8031059

**Published:** 2015-03-12

**Authors:** Gábor Kovács, Zsolt Pap, Cosmin Coteț, Veronica Coșoveanu, Lucian Baia, Virginia Danciu

**Affiliations:** 1 Faculty of Chemistry and Chemical Engineering, Babeș-Bolyai University, Arany János 11, RO-400028 Cluj-Napoca, Romania; E-Mails: gkovacs@chem.ubbcluj.ro (G.K.); ccotet@chem.ubbcluj.ro (C.C.); vcosoveanu@chem.ubbcluj.ro (V.C.); 2 Faculty of Physics, Babeș-Bolyai University, M. Kogălniceanu 1, RO-400084 Cluj-Napoca, Romania; E-Mails: pap.zsolt@phys.ubbcluj.ro (Z.P.); lucian.baia@phys.ubbcluj.ro (L.B.); 3 Faculty of Science and Informatics, Department of Applied and Environmental Chemistry, University of Szeged, Rerrich Béla tér 1, H-6720 Szeged, Hungary; 4 Institute for Interdisciplinary Research on Bio-Nano-Sciences, Babeș-Bolyai University, M. Kogălniceanu 1, RO-400084 Cluj-Napoca, Romania

**Keywords:** titanium-dioxide, aerogels, silver nanoparticles, photocatalysis

## Abstract

TiO_2_-SiO_2_-based nanocomposites with highly porous structures are gaining ever increasing attention due to their specific properties and large variability of synthesis pathways together with wide information on the impact of the synthesis on the activity of the catalyst. This thereby offers an alternative approach to traditional/commercially available photocatalysts. In our work TiO_2_-SiO_2_ based aerogels were obtained and modified with various amount of Ag nanoparticles, using different synthesis pathways. In the first instance their photocatalytic activity was examined in detail, by observing major differences toward salicylic acid and correlating them with their morphological and structural properties (investigating their mesoporous character, band-gap values, crystallinity grade *etc.*). Applying different techniques such as diffuse reflectance spectroscopy (DRS), X-ray diffraction measurements (XRD), transmission electron microscopy (TEM), Raman- and X-ray photoelectron spectroscopy (XPS) the nanoparticles and their composite morphological and structural details were successfully evaluated. Major differences were observed in the activity towards salicylic acid.

## 1. Introduction

The procurement together with the creative combination of well-known materials, while taking into account their functionalities and their properties, is one of the most challenging research-fields in materials chemistry. Considering the alarming level of pollution of the hydrosphere, more and more alternative pathways are being investigated in order to eliminate organic pollutants from the environment. TiO_2_ is considered to be a promising material in the photocatalytic pathway of pollutant-removal, being non-toxic, relatively inexpensive and showing a notable UV light absorption and photocatalytic efficiency toward degradation of various model-contaminants, like phenol [1,2], oxalic acid [3], formic acid [4], and methyl orange [5]. However, like everything in balanced nature, it also has its own insufficiencies which decrease its efficacy. The most representative ones are the wide band-gap energy of native titania, which only allows adsorption of UV light of the solar spectrum and its relatively low active specific surface area (for example, in the case of commercial P25 around 50 m^2^·g^−1^).

One of the solutions for the above mentioned impairments can be the preparation of titania-based aerogels with optimized morphology (surface area and porosity) using the sol-gel method. The main challenge after the “aging” process of the wet gel is to avoid surface tension effects during the drying process and to maintain a porous nanostructure. This can be achieved by freeze-drying, ambient pressure drying with the use of surfactants to lower the surface tension or by the mostly widely used approach, supercritical drying, which can overcome the surface tension effects during the drying process [6]. Regarding titania aerogels, a relatively large number of methodologies have already been published [7,8,9,10,11,12]. The materials generally have higher surface areas than native titania and after a heat treatment, they show high photocatalytic activity, due to an increase in the (photo)catalytically active surface area.

Another approach in the preparation of photocatalytically efficient TiO_2_-based materials is the introduction of another oxide material in order to merge the desired properties. The addition of SiO_2_ to titania can increase the available surface area of the catalyst, allowing an increased efficiency of adsorption of the model-pollutants, thus improving the activity of the composite materials. The properties of the mixed oxides are also strongly dependent on the applied synthesis pathway [13,14].

In the case of obtaining TiO_2_-based photocatalytic materials with higher activity, incorporation of various non-metallic [15] and metallic elements [16] has also been attempted. Noble metals such as gold [17], platinum [18,19], and silver [20] have been highly exploited in this respect. Silver is a favorable metal for TiO_2_-based composites, due to its remarkable electric, optical, and catalytic characteristics [21]. Also Ag nanoparticles have been reported to exhibit high bactericidal activity and biocompatibility compared to other nanoparticles. The synergistic coupling of Ag and TiO_2_ can be attributed to the Schottky-barrier that is formed, giving the possibility for a Ag nanoparticle to act as an electron trap, inhibiting the recombination of photogenerated electron-hole pairs and in this way enhancing the overall photocatalytic activity of the composites [22].

Based on the facts described above, the aim of the present work was the synthesis TiO_2_-SiO_2_-Ag-based aerogels, the investigation of Ag nanoparticles addition effect on their morphological and structural properties using various methods (diffuse reflectance spectroscopy (DRS), X-ray diffraction measurements (XRD), X-ray photoelectron spectroscopy (XPS) and transmission electron microscopy (TEM)), and last but not the least, the correlation of the results with photocatalytic efficiencies obtained on model-pollutant degradation.

## 2. Results and Discussion

### 2.1. Photocatalytic Performance of the Obtained Nanocomposites

As a first step, the photocatalytic activities of the prepared composites were evaluated using salicylic acid, used as a standard pollutant (*C* = 5 × 10^−4^ M). From the slope of the ln *C*_0_/*C*, the kinetic constants of the studied composites were evaluated.

**Figure 1:**
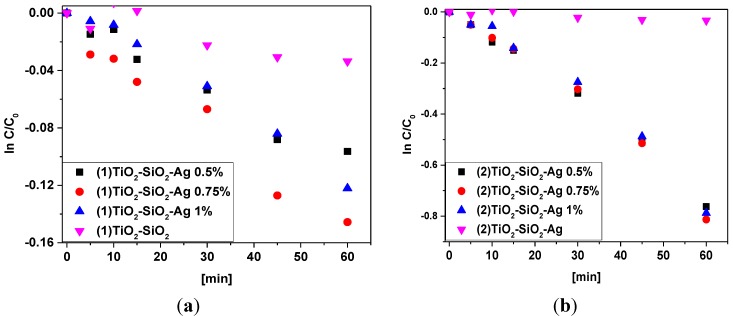
Degradation curves of salicylic acid using the *in situ* preparation method of (1) TiO_2_-SiO_2_-Ag (**a**) and mixing the precursors of TiO_2_-SiO_2_-Ag-based nanocomposites (**b**).

By comparing the obtained photodegradation constants, it can be seen that the composites synthesized by two different pathways show different catalytic behavior: the composites synthetized with impregnation show about five times higher efficiency than composites which have only Ag nanoparticles (Ag NP) in their composition (e.g., 13.07 *vs.* 2.37, apparent kinetic constants obtained using composites with 0.75% Ag concentration). On the other hand it can be observed that the concentration of Ag NP (obtained by synthesis 2, see Section 3.2) does not have a significant effect on the photocatalytic efficacy of the catalysts, the differences between apparent kinetic constants being less than 5% (12.37 × 10^−3^ min^−1^ *vs.* 13.07 × 10^−3^ min^−1^ for the Ag concentrations of 1% *vs.* 0.75% respectively) (Figure 1).

Composites obtained by impregnation have the same tendency if we are looking for the Ag concentration with the highest efficiency; the composite with 0.75% noble metal being the most active (2.37 × 10^−3^ min^−1^). The efficacies decreased significantly in both directions both for increased/decreased Ag content, with 13% and 31% respectively. It has to be mentioned that the unmodified/main catalyst (TiO_2_-SiO_2_) does not have any significant photocatalytic activity; the slight consumption of salicylic acid can be attributed partially to the adsorption of the model pollutant on the surface of the catalyst, a fact that was deducted from the color-change of the catalyst from white to yellow.

### 2.2. Characterization of the Photocatalysts

#### 2.2.1. Morpho-Structural Characterization of the Composites (XRD, TEM)

**Figure 2:**
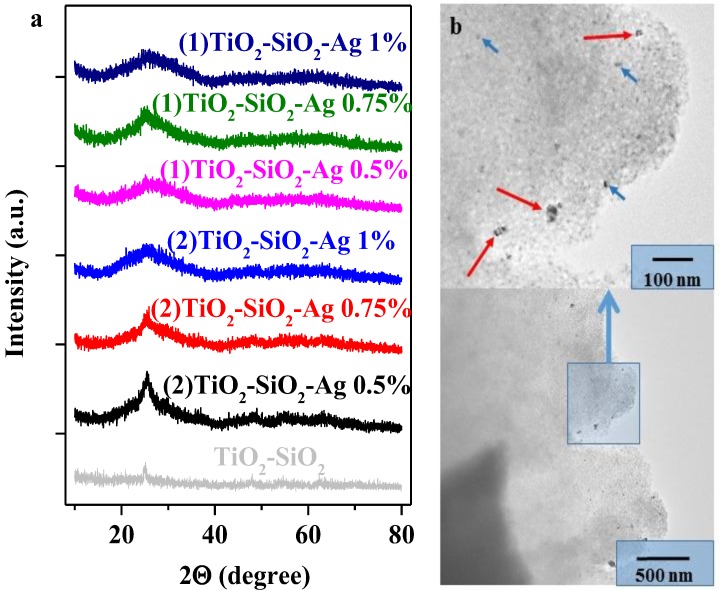
Diffraction patterns of the obtained composites (**a**), and transmission electron microscopy (TEM) micrograph of (2) TiO_2_-SiO_2_-Ag 1% (red arrows show the Ag aggregates, the blue arrows point out the single NPs) (**b**).

As a first step in the characterization process, the crystal size and phase composition was evaluated using diffraction patterns. As shown in Figure 2a, the prepared and thermally treated composites exhibit a weak broad peak around 25.2 °C, suggesting that TiO_2_ is just partially crystallized with a high amount of material still remaining amorphous after thermal treatment, having a crystallization grade around 50% [23]. It can also be observed, that the peak mentioned above is not symmetric, a slight asymmetry of the peak can be observed at ≈30 °C, a fact that suggests the presence of brookite in a small concentration in the structure of the composite, the detail of which was also pointed out by Raman spectroscopy [24].

**Figure A1:**
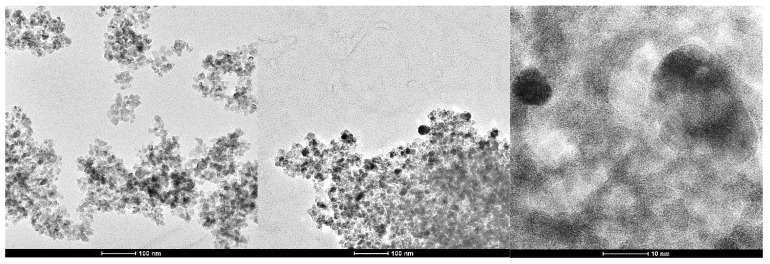
TEM micrographs of (1) TiO_2_-SiO_2_-Ag composites (0.5, 0.75, and 1% Ag content).

In Figure 2b, the TEM micrograph of TiO_2_-SiO_2_-Ag 1% is presented. It needs to be mentioned that this sample was selected to be shown in detail because of the higher Ag concentration, which makes the observation of the isolated Ag nanoparticles and the Ag NP aggregates easier (additional TEM images are presented in the Appendix, Figure A1).

#### 2.2.2. Diffuse Reflectance Spectroscopy (DRS)

Another critical parameter, from the point of view of photocatalytic activity is the light-absorption properties of the TiO_2_-SiO_2_-based composites.

**Figure 3:**
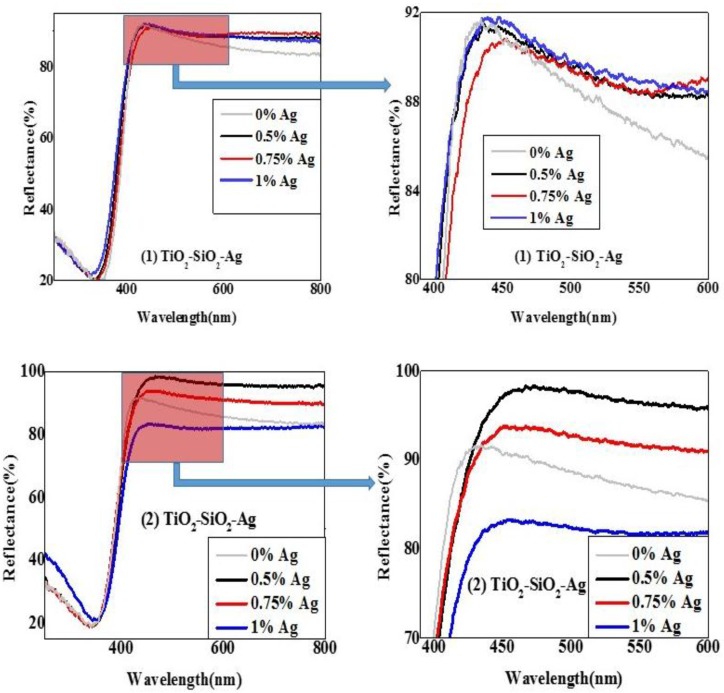
Comparison of different diffuse reflectance spectroscopy (DRS) spectra of the TiO2-SiO2-Ag composites containing differently shaped Au NPs.

**Figure A2:**
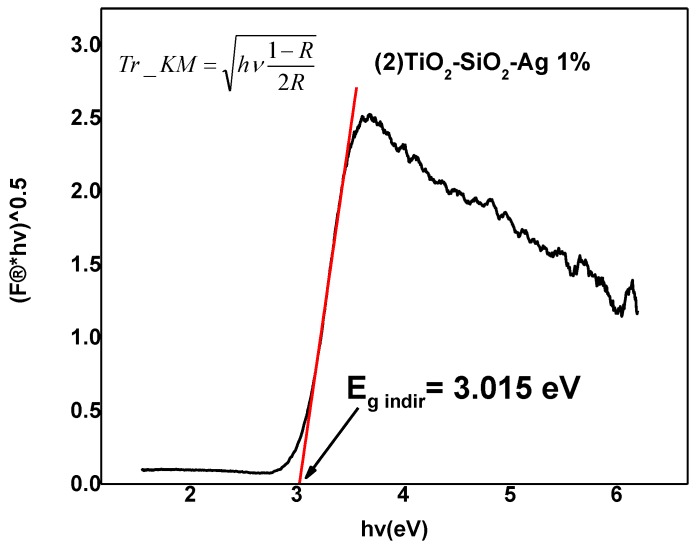
Kubelka-Munk transformation of reflectance spectra regarding the indirect band-gap value of (2) TiO_2_-SiO_2_-Ag 1%.

As can be observed in Figure 3, the differences between the two approaches of the synthesis are clearly visible from the reflection spectra. Consequently, the evaluation of the band-gap values was mandatory. Using the Kubelka-Munk equation, it was found that the second synthesis pathway, (the precursor of Ag was added to the mixture of the precursors of the semiconductor) has both lower direct and indirect band-gap values (3.23–3.30 *vs.* 3.30). The method using TiO_2_-SiO impregnation was not so efficient when taking into account the procurement of composites with lower band-gap values. A clear tendency was not observable, the values obtained being in the range of the reference TiO_2_-SiO_2_. For detailed methodology regarding data evaluation using the Kubelka-Munk equation, please consult the Appendix, Figure A2.

**Table 1:** Photocatalytic efficiencies and morpho-structural properties of TiO_2_-SiO_2_-based composites.

Sample	*k*_app_ × 10^−3^ (min^−1^)	*E*_gdir_	*E*_gindir_	Crystallites mean size [25]
TiO_2_-SiO_2_	-	3.30	2.99	3
(1) TiO_2_-SiO_2_-Ag 0.5%	2.07	3.35	3.08	2
(1) TiO_2_-SiO_2_-Ag 0.75%	2.37	3.27	2.88	3
(1) TiO_2_-SiO_2_-Ag 1%	1.65	3.38	3.09	2
(2) TiO_2_-SiO_2_-Ag 0.5%	12.37	3.23	2.97	4
(2) TiO_2_-SiO_2_-Ag 0.75%	13.07	3.28	3.01	5
(2) TiO_2_-SiO_2_-Ag 1%	12.73	3.3	3.015	4
P25-Aeroxide	11.89	3.45	3.25	-

#### 2.2.3. Raman Spectroscopy

**Figure 4:**
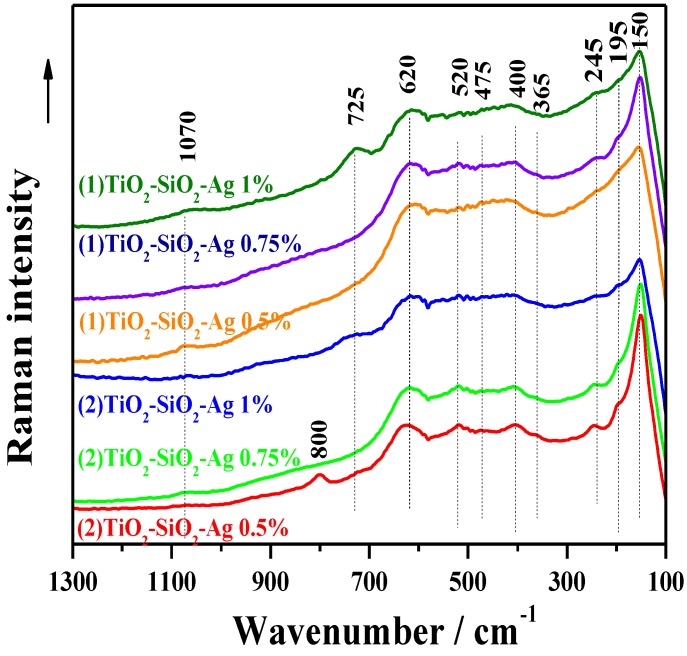
Crystallinity related aspects investigated by Raman spectroscopy.

The Raman spectra are displayed in Figure 4 and show broad features that indicate the existence of a structure whose crystallinity degree is relatively low. The signals around 150, 195, 244, 400, and 620 cm^−1^ are due to the titania vibrations in TiO_6_ octahedral [26,27,28]. Besides the clear signature of the anatase phase (the signals around 195 and 400 cm^−1^ and partially the bands at 150 and 620 cm^−1^ which are most probably convoluted with others originating from another titania phase), the other spectral features around 245, 365 and 620 cm^−1^ (the last is the convoluted one) can be observed. These are probably due to the rutile or brookite phase [28,29].

Keeping in mind that anatase is the most thermodynamically stable phase at sizes less than 11 nm, brookite at crystal sizes between 11 and 35 nm, and rutile at sizes greater than 35 nm [30] and taking into consideration that the structure of the present investigated samples is built up from crystallites of only a few nanometers (see the XRD patterns and the derived data presented in Table 1), the most plausible hypothesis is that the brookite accompanies the anatase phase for all investigated samples. The other Raman signals that appear around 475 and 1070 cm^−1^ are preponderantly associated with the presence of vibrations in SiO_4_ tetrahedral. Thus, the first signal can be assigned to the symmetric stretching vibrations of Si-O-Si, while the second one to the vibrations of Si-O bonds in SiO_4_ units, containing one non-bridging oxygen. The band at 725 cm^−1^ was obviously only apparent because of the high silver content and can be attributed to the deformation of O–(Si, Ti)–O and/or O–Ti–O in chain or sheet units [31,32]. This result shows the important structural influence of silver even if its concentration is relatively low, *i.e.*, 1 wt%. There is another Raman band that can be observed around 800 cm^−1^ only for one of the samples with the lowest silver content, *i.e.*, 0.5 wt%. This band can be assigned to the symmetric stretching vibrations of Si-O-Si in SiO_4_ tetrahedral [33].

#### 2.2.4. X-ray Photoelectron Spectroscopy (XPS)

Until now several interesting aspects have been discussed regarding these materials; determining crystallinity (XRD), optical properties (DRS) and some morphological features (TEM) and related information. However, catalytic/photocatalytic process “localization” is to do with the surface of these materials. Hence, the surface quality (hydrophylicity [34], surface defects [8], anchored surface groups [35], and contamination with carbon deposits) of the applied catalyst should be considered in each case [36]. This statement is also valid for the photocatalytic processes discussed here.

The X-ray diffraction patterns have already shown that these materials are partially amorphous. Furthermore, in each sample series, Ag nanoparticles were introduced. This is rather important, because in the case of aerogels the presence of a noble metal nanoparticle can act as a local crystallization promoter (e.g., Au [37]). While a specific region of the material crystallizes around the Ag nanoparticle, the boundary zone between the amorphous/crystalline could contain a higher amount of defects.

**Figure 5:**
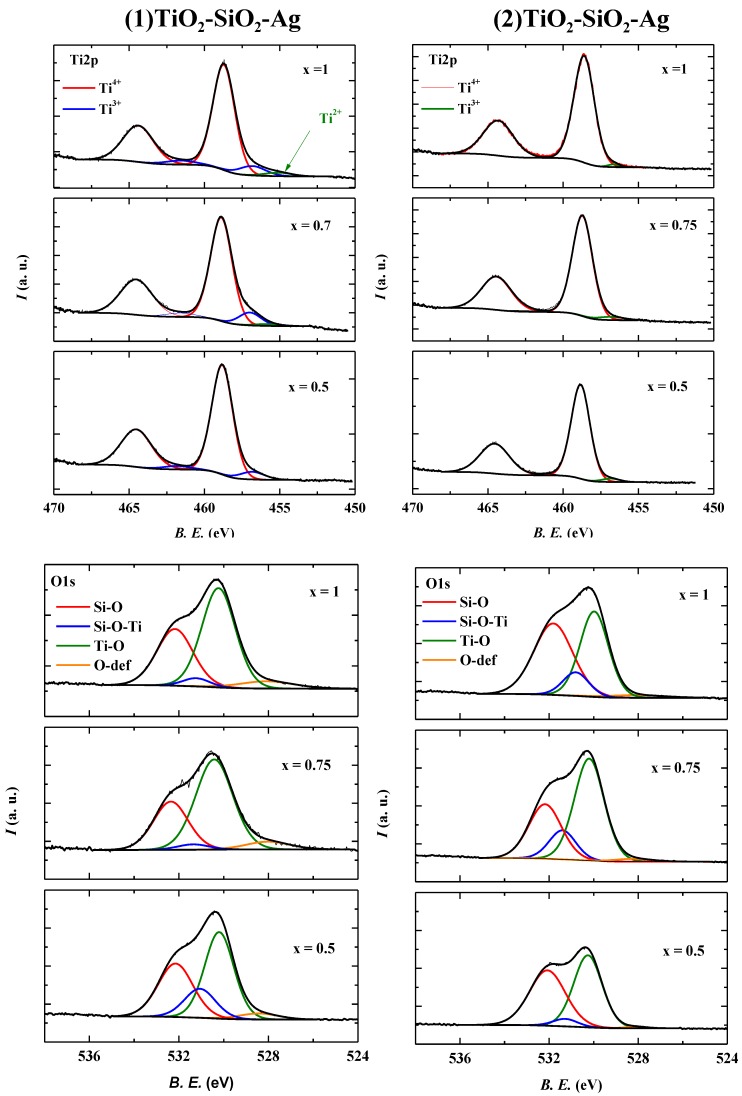

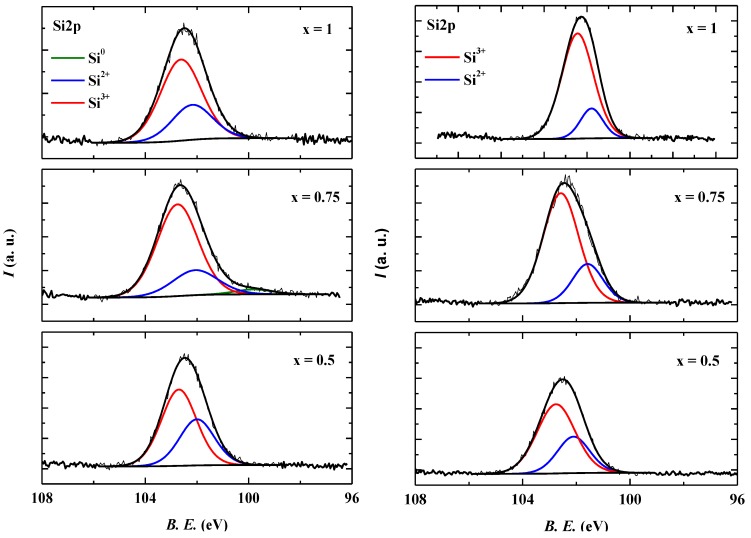
Ti2p, O1s and Si2p XP spectra of the investigated composites.

The Ti2p spectra of the two sample series can be seen in Figure 5. In both cases at 456.8 eV (2p^3/2^) and 461.5 eV (2p^1/2^), the presence of Ti^3+^ [38] was detected, indirectly confirming the presence of the earlier mentioned defects. In sample series (2) TiO_2_-SiO_2_-Ag, the amount of this species was approximately three times lower compared to sample series (1) TiO_2_-SiO_2_-Ag (e.g., 1.13 at% *vs.* 3.18 at% in (2) TiO_2_-SiO_2_-Ag0.5 *vs.* (1) TiO_2_-SiO_2_-Ag0.5). The explanation can be found in the different deposition modes of the Ag. In the case of (2) TiO_2_-SiO_2_-Ag, AgNO_3_ was introduced together with the other precursors (Section 3.2), while in the case of sample series (1) direct reduction of AgNO_3_ with NaBH_4_ was performed in the presence of the already obtained TiO_2_-SiO_2_ aerogel. If the Ag precursor was already in the synthesis mixture, then in the obtained gel/aerogel network (pores) the Ag ions could have been easily reduced, resulting in a “bulk” Ag deposition; this was possible because the pores of the aerogels were larger than the Ag nanoparticles. As XPS is applicable only to the surface of the materials, the Ag nanoparticle driven crystallization effects occurring in the deep-bulk cannot be “seen”, resulting in a significantly lower Ti^3+^ concentration.

If the above mentioned fact is true, then other “clues” for defective growth of this system should be observable. In the case of the O1s XPS spectra, a low binding energy oxygen was noticed in both of the sample series at 528 eV (besides the well-known components of Ti-O at 530 eV and Si-O at 532.2 eV), which, according to our recent work can be correlated with the concentration of Ti^3+^ [8,36,39].

Also in the present case the Ti^3+^ content is directly related to the presence of oxygen vacancies (defects). The defective nature of the sample series (1) TiO_2_-SiO_2_-Ag can also be discussed in the frame of the presence of Ti-O-Si bonds, visible at 531.2 eV [40]. As the concentration of the Ag nanoparticles increases, the concentration of the Ti-O-Si bond decreases from 16.4–3.7 at%. It was known from the DRS spectra that the Ag nanoparticles tended to agglomerate at higher concentration values, resulting in more stable Ag nanoparticle agglomerates. This means that the number of individual particles contacted with the surface of the aerogel is lower with increasing Ag nanoparticle concentration.

When an Ag nanoparticle is in contact with the surface of a semiconductor oxide, the Ag oxidizes slowly, using the O atoms available at the surface (it has already been shown, that a low Ag^+^ concentration can always be found in well-dispersed TiO_2_/Ag systems [41]). This phenomenon forces the Ti and the Si atoms to form the already mentioned Ti-O-Si bonds. Consequently, it is rather simple to imagine that the formation of Ag agglomerates reduces the “use” of surface O atoms, while lowering the probability of Ti-O-Si bonds. The situation is reversed in the case of sample series (2) TiO_2_-SiO_2_-Ag. The Ag nanoparticles are dispersed within the aerogel matrix. Consequently, they cannot form agglomerates, as in the previous case. Hence the concentration of the Ti-O-Si bond increases with the Ag concentration (from 4.3–18.3 at%).

The trends observed until now are also visible in the Si2p spectra and coincide with the ones established in the section discussing the Ti2p and O1s spectra of these materials. In both sample series Si^4+^ (Si2p^3/2^ 103.6 eV; 104.2 eV Si2p^1/2^), Si^3+^(Si2p^3/2^ 103.6 eV; 104.2 eV Si2p^1/2^) and in one case Si^+^(Si2p^3/2^ 103.6 eV; 104.2 eV Si2p^1/2^) was detected [40,41]. Si^3+^ appeared in higher concentration in the sample series (1) TiO_2_-SiO_2_-Ag, while Si^+^ was evident only in (2) TiO_2_-SiO_2_-Ag [42].

#### 2.2.5. The Activity Governing Structural Elements of the TiO_2_-SiO_2_-Ag Ternary Composite Materials

The key to the activity of these ternary nanocomposites resides in several structural features. However, in the present case the main parameter which shows clear signs to be related to the activity is the Ti^3+^ content of the samples. To get important insights regarding this issue the following two important facts should be taken into consideration:

(a) Sample series (1) performed more efficiently in the photocatalytic degradation of salicylic acid under UV irradiation compared to composite set (2): in set (1) the Ti^3+^ content was much higher than in sample set (2)—e.g., 3.18 *vs.* 1.13 at%. Furthermore, in sample set (2) the Ti^3+^ amount is constant, while the activity remains also unchanged (Figure 1, emphasizes the direct correlation of the activity with Ti^3+^).

(b) No clear correlations were established between the photocatalytic activity/crystallinity related aspects or optical properties (band-gap values—Ti^3+^ content).

The two points listed above suggest that indeed the key is in the concentration of Ti^3+^. If the Ti2p spectrum (Figure 5) is examined more closely it can be seen that the sample (1) TiO_2_-SiO_2_-Ag 0.5 contains 3.18 at% of Ti^3+^. As the amount of the reduced Ag nanoparticles increases to 0.7 wt%, the Ti^3+^ concentration starts to increase also. This value reaches 3.82 at% in sample (1) TiO_2_-SiO_2_-Ag 0.7, while the activity is maximized (Table 1). If the Ag content achieves the critical value of 1 wt%, the Ti^3+^ content decreases somewhat (3.52 at%), while Ti^2+^ (0.41 at%) appears as shown by the component Ti2p component located at 455 eV [43]. This extremely reduced species can be easily oxidized to Ti^4+^ by molecular O_2_ (which is also present during the photocatalytic measurements, as described in the Experimental Section), resulting in an overall lower Ti^3+^ content and photocatalytic activity compared to sample (1) TiO_2_-SiO_2_-Ag 0.7.

## 3. Experimental Section

### 3.1. Materials

All chemicals used were of analytical grade. Titanium isopropoxide (TIP), tetraethyl orthosilicate (TEOS), sodium borohydride, nitric acid and silver-nitrate were purchased from Sigma-Aldrich.

### 3.2. The Synthesis of the TiO_2_-SiO_2_-Ag-Based Composites

In order to obtain the desired nanocomposites, two synthesis pathways were followed as below:

1. Synthesis pathway, by *in situ* impregnation of TiO_2_-SiO_2_ aerogels with the Ag precursor (AgNO_3_ dissolved in EtOH) followed by chemical reduction;
2. Mixing the gel precursors for TiO_2_-SiO_2_-based composites with AgNO_3_.

The aerogel used for (1) and for reference material was obtained as follows: a mixture of EtOH:H_2_O_dist_:HNO_3conc_ (16.4:3:0.21 mL) was added dropwise to the mixture containing the semiconductor precursors (TIP:TEOS:EtOH—8.37:3.20:1.7 mL), under vigorous stirring. When the last drops were added, the jellification process started, obtaining the TiO_2_-SiO_2_ gel. The gels were kept in tightly closed polyethylene boxes for 2 weeks for maturation. The impregnation of TiO_2_-SiO_2_ gels with the Ag precursor was made by immersion in 14/21/28 mL of AgNO_3_ (ethanolic solution, 5 mM) for Ag NP concentration of 0.5/0.75/1% and stirred for 2 h. Then, cooled solution (≈4 °C) of 10 mM NaBH_4_ (210/315/420 mL) was added dropwise and stirred for another 2 h. After the impregnation process, the gels were washed three times with EtOH and dried under low temperature supercritical conditions.

Type (2) aerogels were prepared using three different solutions: A–mixture of the semiconductor precursors (8.34 mL TIP, 3.13 TEOS, 22.3 mL EtOH); B–3 mL H_2_O, 0.21 mL HNO_3conc._; C–2.8/4.2/5.6 mL of AgNO_3_ (50 mM ethanolic solution) mixed with 5/7.5/10 μL of H_2_O and 8.37/6.97/5.55 mL of EtOH to obtain 0.5/0.75/1% of Ag concentration. Solution C was added to B and the mixture was added dropwise to A (both under vigorous stirring). The jellification process was observed with the addition of the last drops of solution of B + C. After 2 weeks of maturation, the gels were washed three times with EtOH and dried in supercritical conditionswith liquid CO_2_ (*T* >35 °C, *p* >1200 psi) by using a SAMDRI-PVT 3D (Tousimis, Rockville, MD, USA) equipment. The as prepared aerogels were subjected to a thermal treatment at 600 °C for 2 h (*v* = 4 °C/min).

### 3.3. Characterization Methods and Instrumentation

A Heraeus type photoreactor system with a TQ-150 high pressure mercury lamp (λ >310 nm) was used to measure photocatalytic activity. The photocatalyst suspension containing salicylic acid (*c*_0, substrate_ = 0.5 mM, *c*_photocat_ = 1.0 g/L, *V*_susp_ = 400 mL) was continuously purged by air in order to maintain a constant concentration of dissolved oxygen during the irradiation. The concentration decrease of the chosen pollutant was followed using a JASCO-V650 (Tokio, Japan) spectrophotometer (λ = 297nm).

X-ray diffraction (XRD) measurements were performed on a Rigaku (Prague, Czech Republic) diffractometer (λ_Cu Kα_ = 0.15406 nm, 40 kV, 30 mA, in the 20–40° (2Θ) regime). The crystallites average size was calculated using the Scherrer equation [43].

Transmission electron microscopic (TEM) measurements were executed to characterize the particle size and to identify the morphology of the particles. The TEM micrographs were recorded on a Philips CM 10 (Amsterdam, The Netherlands) instrument operating at 100 kV using Formvar coated copper grids.

A JASCO-V650 spectrophotometer with an integration sphere (ILV-724) was used for measuring the DRS spectra of the samples (λ = 250–800 nm). To obtain the band-gap energy the reflectance data were converted to F(R) values according to the Kubelka-Munk theory. The band gap was obtained from the plot of [F(R)·E]^1/2^ *versus* energy of the exciting light [34,39].

XPS measurements were performed on a SPECS PHOIBOS 150 MCD instrument (Berlin, Germany), with monochromatized Al Kα radiation (1486.69 eV) at 14 kV and 20 mA, and a pressure lower than 10^−9^ mbar. Samples were mounted on the sample holder using double-sided adhesive carbon tape. High-resolution Ti2p, O1s, and Si2p spectra were recorded in steps of 0.05 eV for analyzed samples. Analysis of the obtained data was carried out with Casa XPS software (Cheshire, UK). All peaks were deconvoluted using Shirley background and Lorentzian–Gaussian line shapes. The applied value of the Gaussian–Lorentzian ratio was 30.

## 4. Conclusions

The present study shows a systematic overview and comparison between TiO_2_-SiO_2_-Ag aerogels, obtained by different synthesis pathways. The differences between the photocatalytic efficiencies were correlated with the shift of the material’s optical properties and the structural variations/differences induced by the synthesis routes. It was shown that adjusting the synthesis route and the way in which the Ag NP’s are inserted in the structure of the composite can influence in a more efficient way the photocatalytic activity toward salicylic acid than simply increasing the Ag Nps concentration. This can be attributed to the fact that at higher Ag concentration, the noble metal nanoparticles concentrate in aggregates making their influence/efficiency lower than expected.
